# The effect of brief exposure to virtual nature on mental wellbeing in adolescents

**DOI:** 10.1038/s41598-023-44717-z

**Published:** 2023-10-18

**Authors:** Matthew Owens, Hannah Bunce

**Affiliations:** 1 Department of Psychology, The Mood Disorders Centre, University of Exeter, Exeter, EX4 4QQ UK; 2 The ROWAN Group, Exeter, UK; 3 Somerset Foundation Trust NHS, Taunton, TA1 5DA UK

**Keywords:** Health care, Medical research

## Abstract

Adolescence is a time of multiple transitions and a vulnerability period for mental health difficulties. There are many barriers to the treatment of mental health conditions which is one reason for developing alternatives to help improve efficacy in treatment and prevention. One approach is to use nature-based interventions (NBIs) to improve mental wellbeing. In this experimental proof-of-principle intervention study, we randomly allocated a sample of adolescents to brief exposure (6 min) to either a virtual woodland nature video or a busy train journey and tested the effect on mental wellbeing. Results showed beneficial effects in the nature condition on several self-reported outcomes including stress, relaxation, affect, mood, attention, nature connection and nature spirituality. The intervention was mainly acceptable and feasible to do suggesting that overall brief virtual nature interventions may have utility in a range of mental health contexts for adolescents including as self-help universal or targeted prevention strategies, adjunct to psychological therapy and as preparation for more intensive NBIs. Additionally, brief virtual nature interventions support accessibility for those who may be limited on time, unable to access real-life nature or who may be more *biophobic.*

## Introduction

Mental health difficulties are associated with high levels of disability globally^1,2^ and have huge economic costs attached; approximately £118 billion annually in the UK^3^. Depression, one of the most common disorders, is highly prevalent and on the rise globally^4^. Depression is highly recurrent over the lifecourse, with 50% of sufferers experiencing one or more episodes^5^, and it commonly emerges in adolescence and young adulthood. The age of adolescence has typically been defined as starting with puberty and ending with adult independence^6^ yet there is less consensus on its precise endpoint. While the World Health Organization defines adolescence as spanning the age range of 10–19^7^, it has been argued that expanding this to 10–24 is more meangingful^8^. Adolescence is a time of multiple developmental transitions including cognitive, emotional, physiological and social^9^, making it a period of vulnerability for mental health difficulties. In addition, the negative sequelae of adolescent mental health problems in later life are many including risk for mental health diagnoses^10^, poor educational outcomes and employment prospects^11,12^, poor physical health and social functioning^13^. Furthermore, adolescents are sensitive to stress^14,15^ and stress in this group may be increasing^16^ . It is perhaps unsurprising, therefore, that around 50% of mental disorders appear in the teenage years^17^ and 75% by the age of 24^18^, leading some to highlight the age range of 12-25 as being a key window for early intervention and prevention in tackling depression^19^.

Traditionally, mental healthcare has been driven by our collective ‘rule of rescue’, focusing primarily on treatment^20^. Unfortunately, however, most individuals in need of treatment do not receive any^21^. For those that do seek and receive treatment, many get benefit but it is noteworthy that both in adolescence and across the lifespan, efficacy is limited, typically lower than 50%^22,23^. For example, in reviews of psychotherapy for depression more that 60% of children and adolescents ^24^ and over 50% of adults^25^ did not gain benefit to treatment. Similarly with pharmacological treatments in adolescents, research using treatment-placebo comparisons has shown very small benefit for antidepressants over placebo^26^. Furthermore, a large U.S. population study of adults found no differences on mental or physical quality of life outcomes when comparing those who used antidepressants with those that did not^27^. It is also interesting to note that despite a number of treatment initiatives in recent years we have not seen a resulting reduction in depression prevalence, or the burden associated with it^28^, which has been exacerbated by the response to the COVID-19 pandemic^29,30^. Compounding the reliance on treatment-only models, is the fact that mental health services are currently under strain with unprecedented demand^31^.

For these reasons more focus on prevention interventions has been called for in many quarters^32^ and one area with potential that is under-researched is contact and connection to natural environments^3^, which are thought to be beneficial for human health and wellbeing^33^. It is known that access to green space in childhood is prospectively related to a lower risk of mental health disorder later in life^34^ and it has been shown that spending more time outdoors is associated with good health and wellbeing^35^. As a society we have become less connected to the natural world with 50% of the global population living in urban areas^36^ and we spend as much as 90% of our time indoors ^37^, leading to the concern that we are increasingly less likely to feel the benefit from the natural world.

Nature-based interventions (NBI) have the potential to alleviate mental health difficulties in young people via both treatment and prevention approaches^3,38,39^. While the precise mechanisms involved are as yet unclear^40^, an over-arching theoretical perspective on the positive nature-wellbeing association suggests that humans have a psycho-evolutionary propensity towards natural environments, or a *biophilia*^41^ , also known as nature connection^42^ , which can lead to a range of benefits for health and wellbeing^33^. Specifically, it is thought nature may confer positive effects via stress reduction^43,44^ and restoration of cognitive and emotional functioning following stress and mental fatigue^45,46^. In addition, affect regulation models have been proposed^47^, including assessing the role of depressive rumination^48^. More recently, the potential importance of spirituality in nature in individual wellbeing has been highlighted, an issue that is seldom covered in the literature^49^. Spirituality in nature may include the search for meaning and purpose in life, desire for harmony, hope for an experience of transcendence or recognising that there is something greater than ourselves, or with reference to that which is beyond ordinary human experience^50^.

NBIs are wide-ranging in scope and may involve full contact with wild natural environments such as in the Japanese art of Shinrin Yoku (forest bathing) ^51^ and may incorporate clinical psychological approaches such as CBT^52^, mindfulness^53^ or blended approaches, for example, combining forest bathing and compassionate mind training^54^. Alternatively, interventions may be brief self-help approaches. For instance, in a sample of adolescents a brief nature-based meditation was shown to reduce depressive rumination and produce clinically significant change in depressive symptoms and mental wellbeing relative to control over a 2-week period^55^. Virtual nature exposure may also positively impact human health and wellbeing although effects may not be as strong as for real-world nature^56^. Nevertheless, it is important to recognise that exposure to real-world nature environments may be difficult or impossible for many to achieve on a regular basis, if at all. Barriers include poor urban planning, lack of disposable income, limited mobility^57^, as well as the symptoms of existing mental health conditions such as fatigue and lack of motivation.

It is also important to recognise that access to natural environment spaces is not equally distributed across the population. First, more deprived areas tend to have less green space access than affluent areas^58^ , as do areas where census data indicates ethnic minorities are the majority^59^. Second, other populations that will have restrictions to natural environments, include prison populations and inpatient physical and mental health patients. Third, because adolescents may feel time-poor due to social influence^60^ , social media use^61^ and pressures of academic study^16^, it may be hard for some young people to consistently achieve the recommended weekly contact with natural environments. Therefore, virtual exposure to nature offers a potential adjunct to real-world exposure to natural environments and the evidence suggests that this can be effective in improving wellbeing.

In the present, proof-of-principle study we tested the effect of a brief nature walk video versus an urban control on several mental wellbeing indices including stress, relaxation, negative and positive affect, mood, rumination and self-reported attention. We also set out to test the effect on nature connection and nature spirituality. The study was designed to test the following specific hypotheses: a brief immersive video of a woodland walk (nature condition) relative to a comparison video of a busy underground train journey (urban condition) will improve (i) mental wellbeing and (ii) a sense of connection to nature. We also tested (iii) whether nature spirituality would increase in the virtual nature condition. We note here that we follow the conceptualisation of non-religious spirituality put forward elsewhere, where spirituality may be seen in connection to oneself, others, nature, as well as to transcendence but may not involve religiosity per se^62^.

This study was reviewed and approved by the Psychology Ethics Committee at the University of Exeter (reference number: 530476) and the experiment was carried out in accordance with the principles outlined in the Helsinki Declaration.

## Results

### Randomisation

We first checked whether there were differences between randomisation conditions on demographic information and the baseline measures. No differences were observed suggesting that the randomisation procedure was successful (see Table 1).

**Table 1:** Means, standard deviations and proportions of baseline characteristics and study measures.

Measure	Nature	Urban	Baseline differences (*p*-value)
	Mean (sd)/proportion	Mean (sd) /proportion	
Age	20.19 (1.08)	20.64 (1.18)	0.09
Sex (% female)	32 (86.49%)	32 (84.62%)	0.82
Ethnicity (% white)	32 (86.49%)	33 (84.62%)	0.82
Grew up (% rural)	20 (54%)	20 (52%)	0.81
PSS	7.11 (2.05)	6.62 (2.39)	0.34
SWEMWBS	23.81 (3.89)	23.46 (3.81)	0.69
PANAS POS	14.11 (4.12)	15.18 (3.46)	0.23
PANAS NEG	8.32 (3.13)	9.41 (4.56)	0.23
BSRI	399.62 (126.64)	391.67 (185.73)	0.83
Stress VAS	58.84 (23.01)	57.90 (25.57)	0.87
NCI	33.59 (5.47)	34.18 (5.56)	0.65
NS	27.73 (6.74)	28.05 (5.04)	0.81

P-values refer to differences between conditions. Tests used were ANOVA for continuous data and χ^2^ tests of association for categorical data.

### The effect of nature exposure

#### Stress & relaxation

**Figure 1:**
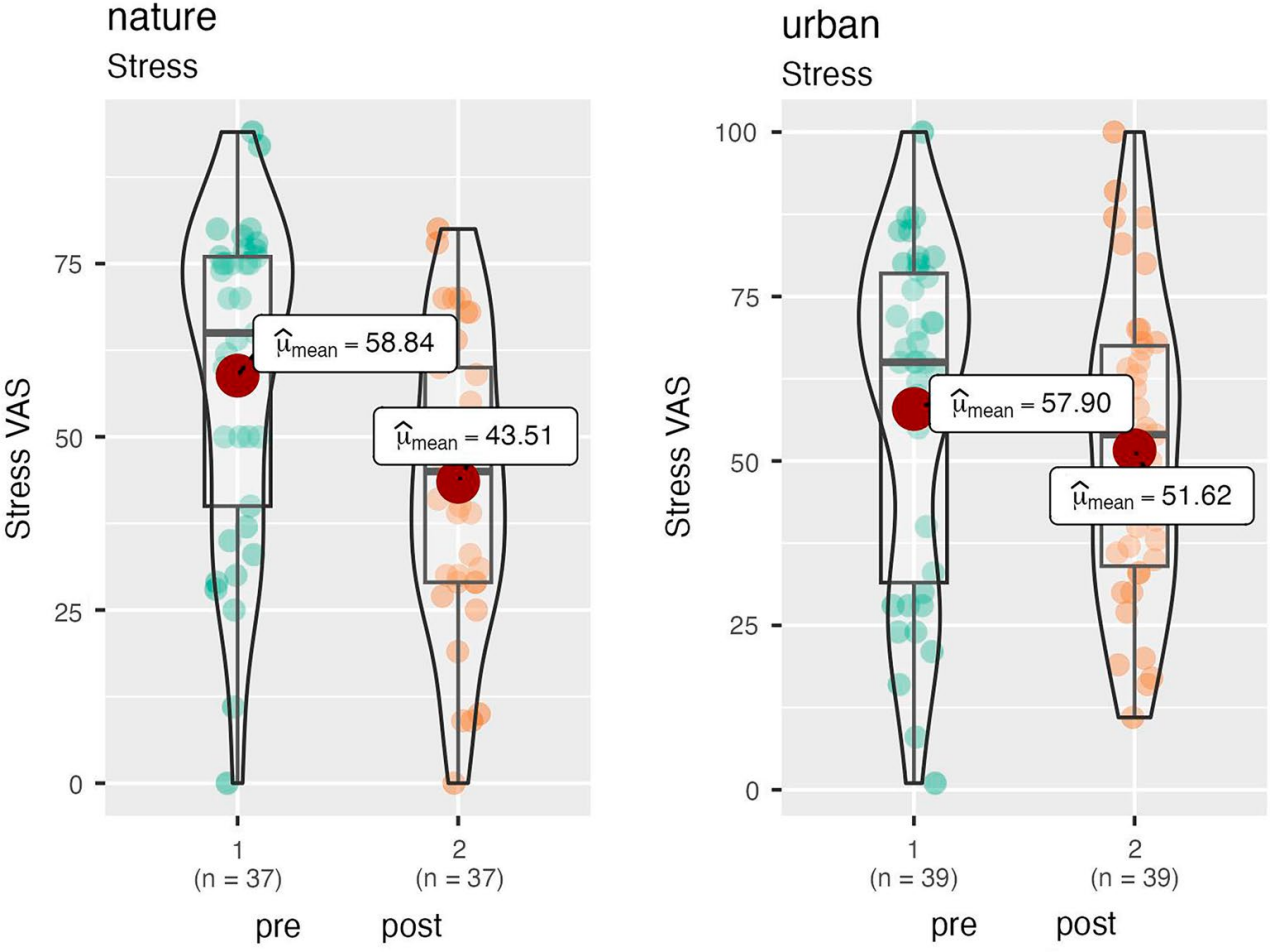
Change in stress over time by nature and urban conditions.

There was a significant effect of Condition on the stress VAS over time (F(1,73) = 4.81, *p* = 0.03, η^2^ = 0.06, marginal mean difference = 8.63; bootstrapped 95%CI = 1.19 to 16.07). Follow-up tests indicated a significant reduction of stress in the nature condition (F(1,36) = 31.02, *p* < 0.001, η^2^ = 0.87) but not in the urban condition (F(1,36) = 2.96, *p* = 0.09, η^2^ = 0.07). Relaxation levels were significantly higher for the nature versus the urban condition immediately following the experiment (F(1,74) = 35.10, *p* < 0.001, η^2^ = 0.32, marginal mean difference = −28.39; bootstrapped 95% CI = −37.60 to −19.17). See Fig. 1.

#### Affect and mood

**Figure 2:**
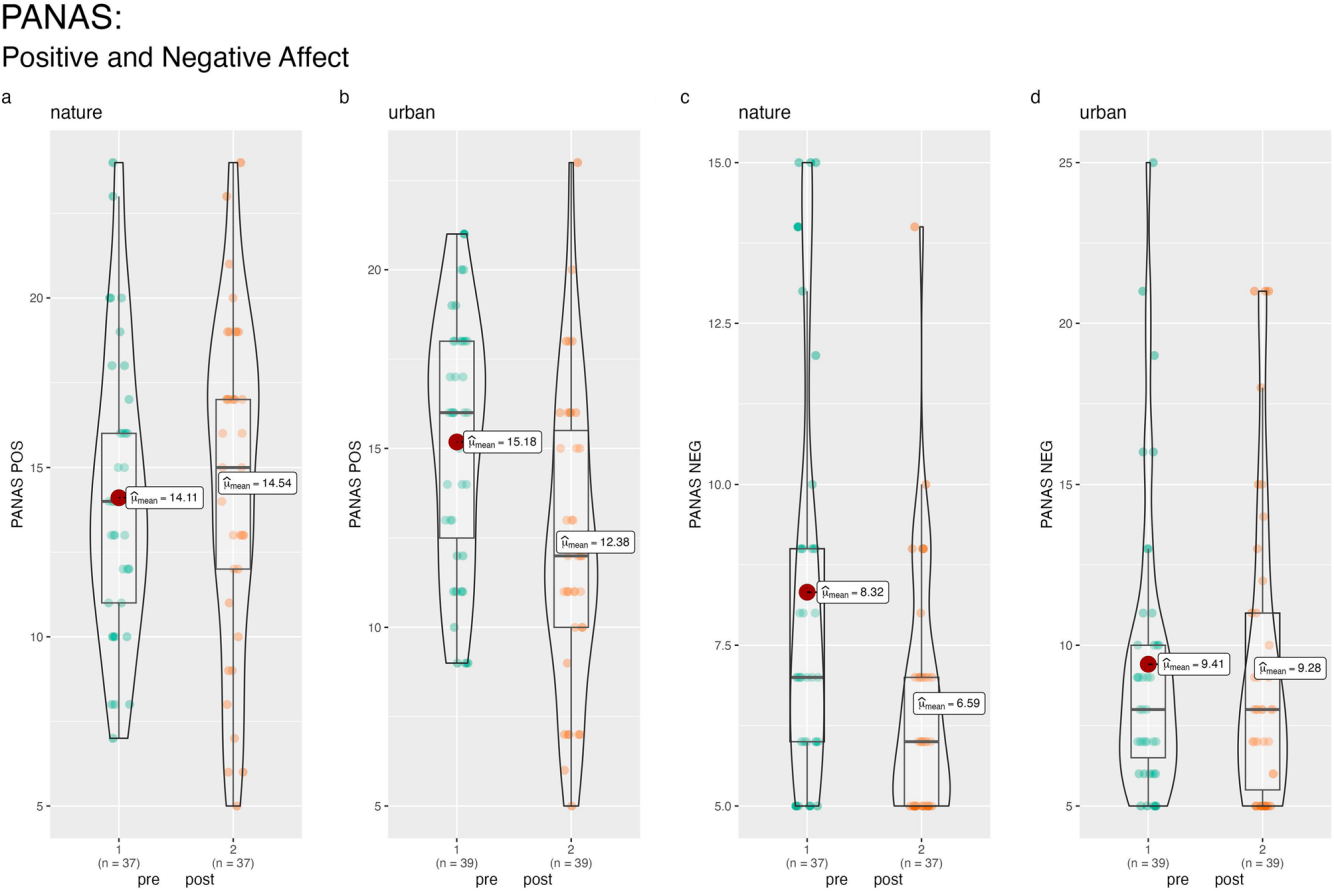
Change in nature positive and negative affect over time by nature and urban conditions.

There was a significant effect of Condition on the PANAS NEG scale (F(1,73) = 11.28, *p* < 0.05, η^2^ = 0.13, marginal mean difference = 1.95; bootstrapped 95% CI = 0.85 to 3.06) and follow-up tests indicated a significant reduction in negative affect in the nature condition (F(1,36) = 26.69,* p* < 0.001, η^2^ = 0.43) and no change in the urban condition (F(1,38) = 0.06, *p* = 0.81, η^2^ = 0.00). Conversely, a significant Condition effect over time for the PANAS POS scale (F(1,73) = 16.20, *p* < 0.001, η^2^ = 0.18, marginal mean difference = −3.02; bootstrapped 95% CI = −4.44 to −1.60) was qualified by a significant reduction in positive affect in the urban condition (F(1,38) = 36.36, *p* < 0.001, η^2^ = 0.49) and no change in the Nature condition (F(1,36) = 0.51,* p* = 0.48, η^2^ = 0.01). See Fig. 2. Positive mood was also rated as significantly higher on the VAS in the nature condition immediately following the experiment (F(1,74) = 25.08, *p* < 0.001, η^2^ = 0.25, marginal mean difference = −23.41; bootstrapped 95% CI = −32.31 to −14.50).

#### Nature connection & nature spirituality

**Figure 3:**
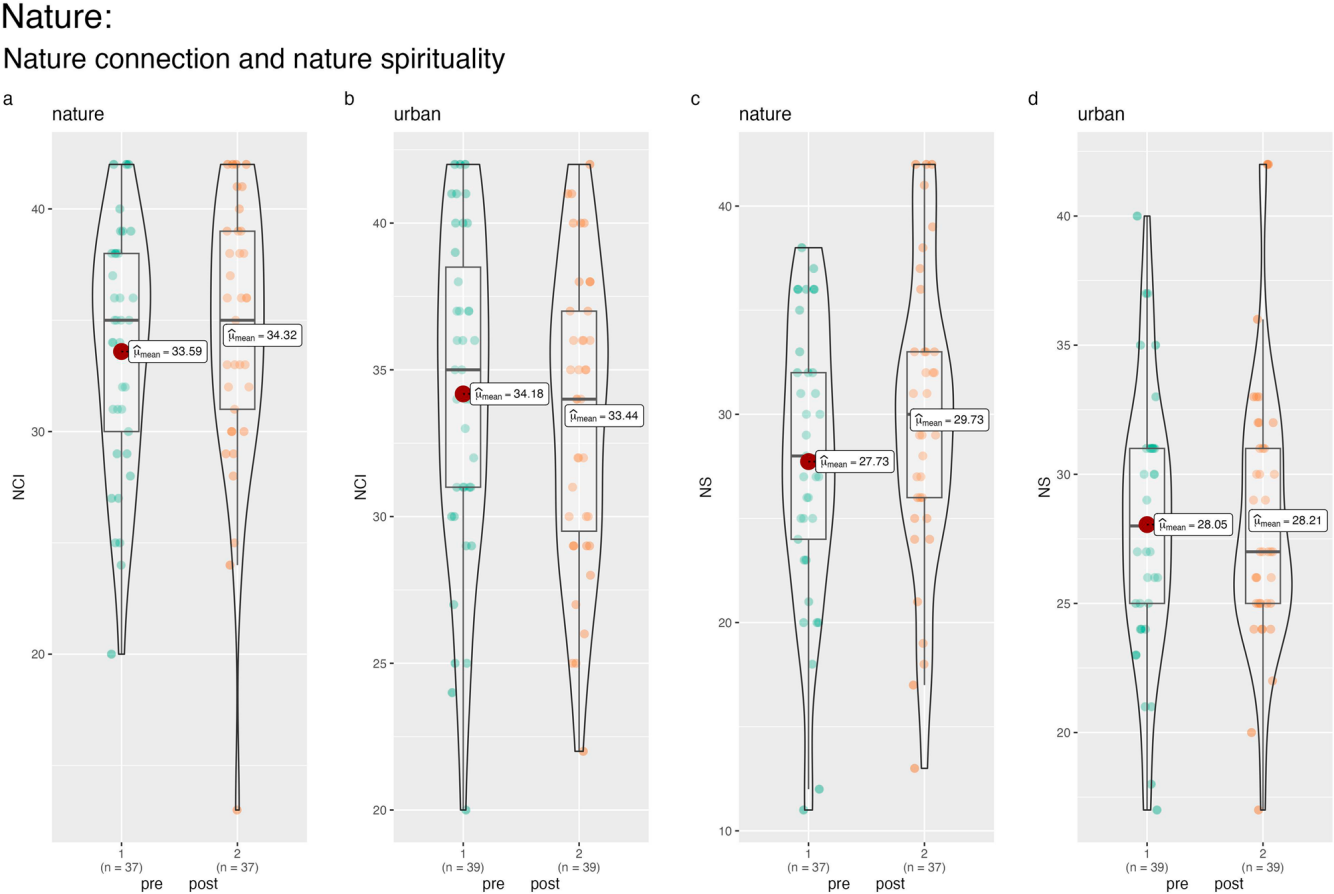
Change in nature connection and nature spirituality over time by Nature and Urban conditions.

There was a significant effect of condition on the NCI (F(1,73) = 5.37, *p* < 0.05, η^2^ = 0.07, marginal mean difference = −1.41; bootstrapped 95% CI = −2.57 to −0.24), where nature connection increased in the nature condition but not significantly (F(1,36) = 3.09,* p* = 0.09, η^2^ = 0.08) and decreased but not significantly in the urban condition (F(1,38) = 2.65,* p* = 0.11, η^2^ = 0.07). Similarly, there was a significant effect of Condition on self-reported nature spirituality (F(1,73) = 8.43, *p* < 0.01, η^2^ = 0.10, marginal mean difference = −1.82; bootstrapped 95% CI = −3.05 to −0.50) which increased in the nature condition (F(1,36) = 15.76,* p* < 0.001, η^2^ = 0.30) but did not change in the urban condition (F(1,38) = 0.16,* p* = 0.69, η^2^ = 0.00). See Fig. 3.

#### Affect regulation

There was no significant difference between the conditions on change in rumination as measured by the BSRI (F(1,73) = 1.96, *p* = 0.17, η^2^ = 0.03, marginal mean difference = 31.25; bootstrapped 95%CI = −13.30 to 75.79)

#### Attention

In the nature condition, participants reported being more focussed and alert immediately after the experiment relative to the Urban condition (F(1,74) = 7.03, *p* < 0.05, η^2^ = 0.09, marginal mean difference = −12.66; bootstrapped 95% CI = −21.62 to −3.70).

### Post experiment questions

#### Attention check

The majority of participants in the nature condition (33, 89.19% correct) and Urban condition (33, 84.62% correct) successfully completed the attention check at the end of the video. There was no difference between the two conditions (χ^2^ = 0.35, *p* = 0.56).

#### Acceptability and feasibility

Twenty-four participants in the Nature condition (64.86%) and 33 in the Urban condition (84.62%) reported that the video felt either ‘slightly realistic’ or ‘very realistic’ when watching it, which was a significant difference (χ^2^ = 3.95, *p* = 0.05). When asked about the length of the videos, participants in the Nature condition reported that it was ‘just right’ (5, 13.52%), ‘a bit long’ (21, 56.76%) or ‘too long’ (11, 29.73%); participants in the Urban condition reported that the video was ‘just right’ (7, 17.95%), ‘a bit long’ (25, 64.10%) or ‘too long’ (18, 23.68%). There was no difference between conditions (χ^2^ = 1.52, *p* = 0.47). When asked whether the videos were helpful for anxiety and depression, 72.22% of those in the nature condition versus 43.59% in the urban condition reported that they would recommend the intervention to a friend or family member experiencing anxiety or depression (χ^2^ = 6.27, *p* = 0.012).

## Discussion

Our findings support both of the general hypotheses that brief exposure to an immersive nature video, relative to an urban comparison condition would (i) reduce stress and improve indices of mental wellbeing in adolescents and (ii) increase nature connection. Our novel hypothesis on increasing nature spirituality (iii) was also supported. The positive findings are consistent with previous research where virtual nature interventions often, but not always, show benefit^63^. For example, in a review of virtual nature interventions including photographs, slideshows, videos and virtual reality, 45% of studies with mood as an outcome produced positive results^64^. Comparing virtual versus real-world nature exposure, a related meta-analysis showed that real-world nature improved mood more than virtual nature but there was no difference on negative affect^56^. However, a recent study comparing the effects of real-world forest bathing with a virtual reality version found beneficial effects on a range of outcomes including mood, stress and affect. Although the pattern in the data suggested larger effect sizes on some outcomes, there were few statistical differences reported between groups^65^.

In the present study, we first showed that the randomisation to the Nature and Urban conditions was successful, with no significant baseline differences found on any measure. This is an important step, and broader methodological issue, as previous work in this area has shown that both lack of randomisation and lack of the use of a control group can inflate the positive finding rate^64^. The study was designed to detect nature effects from a previous real-world study^55^ but lower than expected recruitment reduced our power from 90 to 77%. Nevertheless, we were able to significantly detect differences between the two conditions on the majority of outcomes, with the notable exception of depressive rumination. The null result for rumination in the present study may indeed be a product of low power but it may also reflect the low potency of the virtual nature exposure relative to real world interventions^56^. Reductions in rumination have been demonstrated in other real-world studies^38,55,66^ and so we believe that this is most likely explained by a combination of low power and relatively weaker efficacy of virtual exposure to nature.

To encourage adherence to task in this online remote context, we asked participants a factual checking question specific to each condition. This proved to be helpful given that the majority in each condition answered their respective questions correctly and we suggest continuing to use measures to increase adherence to online interventions in future research.

There was a difference between conditions on positive affect which decreased significantly in the urban condition but not in the nature condition, suggesting that rather than improving positive affect, the nature condition was able to buffer it, relative to the deleterious effects in the urban condition. This may be very important when considering future early intervention and prevention strategies for adolescent mental health, especially for those for whom accessing nature is challenging. Buoying mental wellbeing for those at-risk adolescents may mean the difference between sustaining wellness and facing the unfolding of clinical mental health difficulties.

An increase in nature spirituality in the virtual nature condition is a novel finding that may also be important for future research directions. As has been pointed out by Ryff^49^, despite a long tradition of spirituality in philosophy, literature and culture, spirituality has not thus far featured in conceptualisations of wellbeing and flourishing in the psychological literature. Research suggests that spiritual health is important for young people and is strongly associated with self-perceived personal health^67^ and so should be explored further. Whether nature spirituality is likely to be seen as forming part of our overall wellbeing or whether nature facilitates spiritual wellbeing remains to be seen.

The paucity of spirituality considerations in clinical and counselling training programmes is slowly being addressed^68^ in an attempt to more fully support the whole individual in their context. The inclusion of nature spirituality questions as part of the assessment process may aid general suitability of NBIs and in addition serve to focus the mind into values-based interventions, such as Acceptance and Commitment Therapy (ACT)^69^. Identifying values that are meaningful to an individual has been shown to be helpful in reducing the extent to which individuals are *fused* with their negative thoughts and feelings, through connecting and committing to behaviours that are in service of their values. For some, being asked to reflect on nature spirituality may prompt individuals to reconnect with biophilic values.

We recognise that virtual exposure to natural environments may have value in improving mental wellbeing and even preventing mental illness, and may be appropriate for those struggling to get outside or who are agoraphobic, socially phobic or have depression-related low motivation. However, consistent with other views^57^, we do not suggest that virtual nature exposure should typically be a replacement for the real-world experience. Instead, virtual exposure could be used as a self-help intervention, an adjunct to therapy or reserved for those for whom accessing nature is challenging, whether due to individual factors or systemic inequities. The use of a nature video in therapeutic settings may be one of a variety of relaxation strategies, or ‘tools’ that can be curated for an individual. It could be used throughout therapy, as part of home practice and as relapse prevention packages. It may also support mindful practices for those who require more concrete stimuli to support their practice. Given the ubiquity of personal technological devices, accessibility to watch a nature video may be more available for those who rely on others to take them into real-world nature, as is the case for those whose mobility is compromised or for younger children. This also has the potential to redress some of the power imbalance, with regards to accessing the health benefits of nature. For those who may be *biophobic*, nature videos may be an option for a graded exposure programme to acclimatise to real-world nature and ‘wilderness’ nature, thus supporting them to reap the benefits of more intensive NBIs. Such interventions may be required to effect larger, sustained change or change in maintaining factors of low mood, for example, depressive rumination.

Although just a single subjective item explored the effect of nature on attention, participants self-reported feeling more alert and attentive after watching the video. It should be noted that in both virtual experiments^70^ and real-world settings using objective measures of attention have produced mixed results^46^. Nevertheless, the video could be utilised to support challenging or difficult situations by preparing the individual cognitively to be more alert to the task at hand. In therapy, this could be used to support individuals to increase awareness, a skill that is important across different therapeutic modalities, to spot maladaptive coping mechanisms and patterns in order to choose different responses to a situation, rather than to be reactive.

The majority of the sample reported that the video was too long although we consider it brief, under the average time for virtual exposure (8 min) in a recent review ^64^. Despite this, however, there were positive effects of the virtual nature exposure on most outcome measures. Future research should test the dose–response relationships to find optimal, possibly shorter durations of stimuli presented. Most participants reported that the videos were realistic although this proportion was larger in the urban condition. Again, it is not clear whether this is a crucial factor in the efficacy of intervention, but future research could compare these perceptions with more immersive virtual reality. The majority of participants in the nature condition (72%) reported that they would recommend the intervention to someone in need of help with anxiety of depression.

The strengths of this study include remote blind randomisation of participants to experimental or comparison condition, the use of a number of well-validated psychometric instruments and the novel finding of an increase in nature spirituality following brief virtual nature exposure. We acknowledge, however, several limitations. First, in this proof-of-principle study we are unable to determine the precise features of environment that are likely to be beneficial to mental wellbeing. As discussed elsewhere this is an important frontier in the research ^33^. The experience in real-world natural environments is also multisensory and it is not clear from the present study which modality, or combinations thereof, are most important. In addition, it is important to recognise that there are non-sensory pathways to wellbeing. For example, phytoncides (compounds found in the air in natural environments) may confer a plethora of benefits to the individual including improved immune functioning, improved sleep and decreased stress and anxiety ^71^.

We were unable to use objective measures of stress (e.g. salivary cortisol) or indices such as heart rate variability collected via wearables, or objective measures of attention or other executive functions. We were also unable to include any follow up measurement time points to test, for example, the potential mid-term effect on reducing depressive symptoms over weeks and months. Clearly in this proof-of-principle, experimental design of a brief intervention we were unable to test effects of prevention of mental health conditions in adolescents but this should be a focus of future research. The sample of adolescents in this study was also overwhelmingly female and ethnically white and so it will be important for future studies to test for effects in males and other ethnic groups.

We suggest that future research priorities should focus on augmenting virtual nature interventions to increase their effectiveness and this could be achieved in several ways. First, the intervention could be repeated multiple times over a number of days or weeks and dose-response relationships assessed. Second, more active components could be added, for example, mindful awareness^72^, breathing techniques^73^ or encouraging participants to summarise and record how they felt after the video to support recall of the positive benefits^74^. Third, nature type (e.g. blue, green or brown space^75^) could be presented as a choice for individuals to assess the effect of preference and potentially maximise adherence. Incorporating individual differences may also enhance a sense of nature spirituality and wellbeing. Fourth, research into the optimal length of video for different aged children and adolescents should be explored. Fifth, to understand the most important features and modalities in elements of nature, virtual conditions should be compared with real-world interventions. Lastly, testing the effect of virtual nature exposure as part of a wider package of intervention, including therapy, should be prioritised in order to tackle the burden of mental health difficulties in young people through both prevention and early intervention approaches.

## Methods

### Procedure

The study used an experimental design and recruited participants through opportunity sampling. Recruitment methods included social media (e.g. Facebook, Instagram, LinkedIn), posters placed around the University campus and through word of mouth. Digital and paper advertisements contained a QR code for participants to access study information and a consent form. Once participants had provided informed consent, they completed baseline measures housed in the Gorilla Experiment Builder. Participants were then randomly assigned 1:1 to either the nature or urban condition using the Gorilla randomizer node. This process is automatic and not influenced by the researchers. Participants were shown a brief, 6-min video of either a nature or urban setting and were presented with an attention check question to measure adherence. Participants were then asked to complete the post-experiment self-report battery. At the end of the study, participants were given a full debrief of the study aims and were invited to be kept updated with the study results. Signposting to mental health services was also provided. Lastly, participants were automatically entered into a prize draw for the chance to win one of three possible cash voucher prizes (1 × £30, 2 × £10).

### Participants

The sample consisted of 76 adolescents (mean age = 20.42, SD = 1.15, range = 18–25) and included 61 females (85.53%), 11 males (14.47%). The majority, 65, was white (85.53%) with 11 (14.47%) participants reporting other ethnicities (3 ‘Asian’, 1 ‘Black’, 3 ‘Mixed’ and 4 ‘Other’).

### Conditions

#### The virtual nature condition

This condition contained a short virtual nature video (6 min) combining immersive visual and auditory aspects of both green and blue space. The recording is a point-of-view walk that begins along a woodland pathway surrounded by tall trees, followed by a section of riverside walking in the woodland, ending with a further pathway walk. Various natural sounds, such as birdsong, the rustling of leaves and water trickling in the river, can be clearly heard in the video. The reasoning for selecting this nature video was to combine both green and blue space and to provide a short but immersive, holistic virtual nature experience that analogous to natural green/blue spaces used in previous real-world research^55^ . Potential benefits of elements in the recording such as woodlands ^76^ , sounds of water^77^ and birdsong ^78,79^ are all underpinned by the evidence base.

#### Urban comparison condition

**Figure 4:**
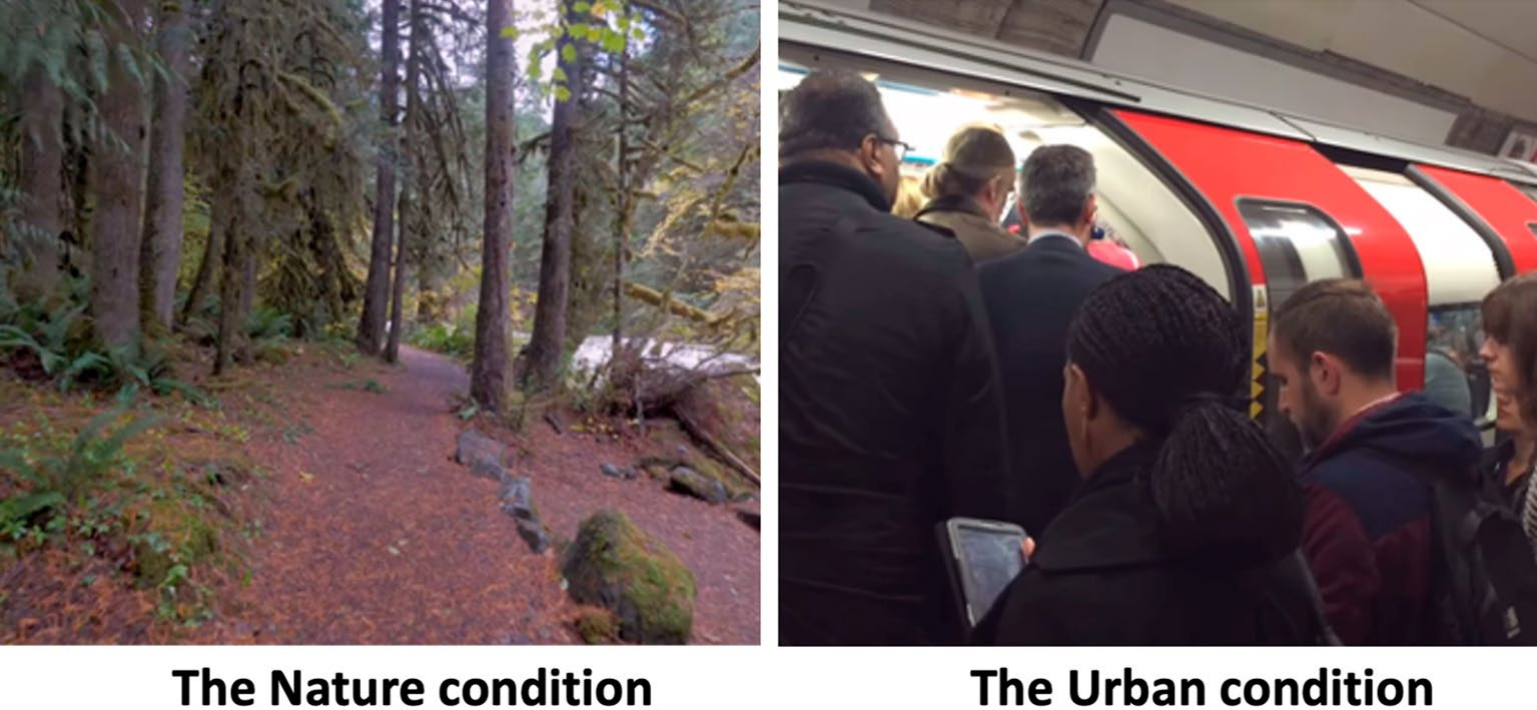
Stills from the videos in the Nature and Urban conditions.

This comparison condition included a recording of equivalent duration to the virtual nature condition (6:00 min) that attempted to emulate a real-world urban environment. The video is taken inside a London underground train during a busy rush hour period. The video features a virtual passenger waiting at a busy and congested platform for a London Underground train to arrive. The train arriving is full of commuters and features lots of people trying to board the train. Upon entering the train, the commuter stands for the rest of the duration of the video, at close proximity to the ceiling. Noisy urban sounds can be heard throughout the video such as loud engine sounds of the trains travelling, arriving and departing from various stations, commuters talking and public service announcements being made. The experience in this recording involves exposure to multiple potential urban environmental stressors through virtual immersion in a noisy, crowded setting which in the real-world may increase aversive emotional responses ^80^, stress ^81^, anxiety, depression^82^, sleep disturbance ^83^ and be associated with somatic health problems, potentially mediated by cerebral changes ^84^. See Fig. 4 for video stills for each condition.

### Measures

#### Short Warwickshire Edinburgh Mental Wellbeing Scale (SWEMWBS)

The SWEMWBS is a seven-item unidimensional measure of wellbeing^85^ that has demonstrated utility in its brevity, reducing participant burden and avoiding gender bias when compared with the full 14-item version^86,87^. Participants are asked to think back over the past two weeks and respond to items, including “I have been feeling optimistic about the future”, using a five-point Likert scale (1 = “none of the time” to 5 “all of the time”) with scores ranging from 1 to 35. The SWEMWBS has shown good validity^85,88^ and reliability (α = 0.86–0.88^88,89^). Reliability was good in the current study (α = 0.76).

#### International Positive and Negative Affect Schedule Short Form (I-PANAS-SF)

The I-PANAS-SF is a 10-item scale measuring state positive affect (PA) and negative affect (NA) using a five-point Likert scale (1 = “not at all/ very slightly to 5 = “extremely”)^90^. Participants are asked to reflect on how strongly they feel each item *at the moment* (for example, “determined”, “nervous”). The I-PANAS-SF has demonstrated good factorial invariance across different cultures^91^ and good validity and reliability (PA α = 0.78 and NA α = 0.76^90^). In the current study reliability was good at baseline, PA α = 0.71, NA α = 0.83 and post intervention, PA α = 0.84 and NA α = 0.89.

#### Brief State Rumination Index (BSRI)

The BSRI is an eight-item scale measuring state rumination using a visual analogue scale (VAS; 0 = “completely disagree” to 100 = “completely agree”)^92^. Participants were asked to reflect on items *in the moment* (for example, “Right now, I wonder why I always feel the way I do”). The scale has demonstrated good validity and reliability (α = 0.91)^92^. Reliability was good in the current study at baseline (α = 0.86) and post intervention (α = 0.90).

#### Stress

State stress levels were measured using a VAS (0 = “completely disagree” to 100 = “completely agree”), asking participants to rate how strongly they agree with the statement “I am stressed right now”. VAS stress scales have been shown to be equal to questionnaire methods of assessing stress^93^ and correlate with scores on the Perceived Stress Scale (PSS^94^; *r* = 0.65, *p* < 0.001) and havedemonstrated predictive power for the PSS^95^.

#### Perceived Stress Scale (PSS-4)

The PSS-4^96^ is a four-item scale measuring perceived psychological stress in the last month, using a five-point Likert scale (“Never” to “Often”). Participants were asked to respond to questions about the frequency of thoughts and feelings relating to stress (for example, “In the last month, how often have you felt that things were going your way”). The scale has shown good validity and reliability^97,98^ (α = 0.64 in the current study) and has utility in its brevity for administration^97,99^, despite a reduction in internal reliability from the original 14-item scale (α = 0.60 vs 0.85^100^).

#### Nature connection

The Nature Connection Index^101^ (NCI) is a unidimensional scale measuring nature connectedness in children and adults using six items on a seven-point Likert scale (1 = completely disagree to 7 = completely agree). Items include “I always find beauty in nature” and “Spending time in nature is very important to me”. The scale has demonstrated good validity and reliability in previous research ^101,102^ and was good in the present study at baseline (α = 0.89) and post intervention (α = 0.90).

#### Nature spirituality

For the present study, a small set of items was needed to test our exploratory hypothesis on nature spirituality in adolescents whilst not overburdening participants. To ensure that items were likely to be tapping into state spirituality and therefore sensitive to change after a short duration, we used the stem of “Right now,…” for each item. We selected items we believed would be most appropriate for this sample of adolescents experiencing a virtual nature scene. We selected four items from the 20-item Ecospirituality Scale^103^ and combined them with two further bespoke items. Participants were asked to indicate how on a 7-item Likert scale the extent to which they agreed with each statement (“completely disagree” to “completely agree”). An example item includes, “Right now, it gives me pleasure to see the beauty of life in this universe”. Scores over the six items were summed to create a total score. The six-item battery demonstrated good reliability (α = 0.78 at baseline and 0.84 post intervention) and the test–retest reliability was good (*r* = 0.89).

#### Bespoke items

Three additional items were included following the experiment. These were “Right now, I feel relaxed/composed”. “Right now, I feel that my mood is positive”. “Right now, I can focus clearly and I feel alert”. Each item used a VAS (0–100).

#### Feasibility and acceptability

We asked three questions at the end of the experiment to assess feasibility and acceptability of the intervention, including, “Overall, how realistic did watching the video feel?”, “How did you find the overall length of the video? And “Would you recommend this to a friend or family member who is experiencing anxiety or depressive symptoms?

#### Sample size calculation

We based our sample size calculation on detecting a difference in short-term psychological change following a nature-based intervention ^55^ (partial eta-squared = 0.11). Using G*Power 3.1, a total sample size of 106 was required to have 90% power to detect this effect size on any of the outcome measures. In the event, we recruited a total number of 76 participants, which had the effect of reducing the statistical power in the study to 77%.

### Statistical approach

We used STATA (version 17) to conduct the statistical analysis. We tested the differences between the randomly allocated conditions on baseline measures using analysis of variance (ANOVA) for continuous data and chi-square tests of association for categorial data. When testing the hypothesis concerning the relative beneficial effect of the nature versus the urban condition, we used analysis of covariance (ANCOVA) with a condition variable as the fixed factor (nature = 1, urban = 2), the post-experiment scores specified as outcomes and the pre-experiment scores entered as continuous covariates. In this way, the relative change from pre to post experiment can be assessed in a statistically powerful model^104^. Following the ANOVA/ANCOVA models, we used bootstrapping to estimate the marginal mean differences on the outcome measures (adjusted for the relative baseline measure in each model, as appropriate). We requested 1000 resamples and report the bootstrapped 95% confidence intervals (C.I.) around the estimates. Unadjusted mean differences are shown in Table 2. For significant effects of Condition, we also ran follow up repeated measures ANOVA tests, within each condition separately, to illustrate the effect of condition on the outcomes. Combined box plots/violin plots were created using the grouped_ggbetweenstats function in R Studio.

**Table 2:** Means and standard deviations of study measures by condition and time.

Measure	Nature		Urban	
	Pre	Post	Pre	Post
Stress VAS	58.84 (23.01)	43.51 (20.71)	57.90 (25.57)	51.62 (22.9)
PANAS POS	14.11 (4.19)	14.54 (4.82)	15.18 (3.46)	12.38 (4.09)
PANAS NEG	8.32 (3.13)	6.59 (1.95)	9.41 (4.56)	9.28 (4.72)
BSRI	399.62 (126.64)	324.81 (121.98)	391.67 (185.73)	350.13 (177.94)
NCI	33.59 (5.47)	34.32 (6.04)	34.18 (5.56)	33.44 (5.05)
NS	27.73 (6.74)	29.73 (7.13)	28.05 (5.04)	28.21 (5.07)
Relaxation	–	71.51 (17.13)	–	43.13 (23.89)
Mood	–	66.43 (18.40)	–	43.03 (22.07)
Attention	–	59.89 (18.63)	–	47.23 (22.68)

*Stress VAS* single item VAS, *PANAS POS* positive affect subscale on the PANAS, *PANAS NEG* negative affect subscale on the PANAS, *BSRI* Brief State Rumination Inventory, *NCI* nature connection index, *NS* nature spirituality, a bespoke measure of nature spirituality, *Relaxation* single item VAS, *Mood* single item VAS, *Attention* single item VAS.
